# Behavioral Studies and Genetic Alterations in Corticotropin-Releasing Hormone (CRH) Neurocircuitry: Insights into Human Psychiatric Disorders

**DOI:** 10.3390/bs2020135

**Published:** 2012-06-21

**Authors:** Gloria Laryea, Melinda G. Arnett, Louis J. Muglia

**Affiliations:** 1 Neuroscience Graduate Program, School of Medicine, Vanderbilt University, 465 21st. Avenue South, Nashville, TN 37232, USA; E-Mail: gloria.n.laryea@vanderbilt.edu; 2 Center for Preterm Birth Research, Cincinnati Children’s Hospital Medical Center, 3333 Burnet Avenue, Cincinnati, OH 45229, USA; E-Mail: melinda.arnett@cchmc.org

**Keywords:** corticotropin-releasing hormone, anxiety, depression, psychiatric disorders, human polymorphisms, CRH receptors, CRH binding-protein

## Abstract

To maintain well-being, all organisms require the ability to re-establish homeostasis in the presence of adverse physiological or psychological experiences. The regulation of the hypothalamic-pituitary adrenal (HPA) axis during stress is important in preventing maladaptive responses that may increase susceptibility to affective disorders. Corticotropin-releasing hormone (CRH) is a central stress hormone in the HPA axis pathway and has been implicated in stress-induced psychiatric disorders, reproductive and cardiac function, as well as energy metabolism. In the context of psychiatric disorders, CRH dysfunction is associated with the occurrence of post-traumatic stress disorder, major depression, anorexia nervosa, and anxiety disorders. Here, we review the synthesis, molecular signaling and regulation, as well as synaptic activity of CRH. We go on to summarize studies of altered CRH signaling in mutant animal models. This assembled data demonstrate an important role for CRH in neuroendocrine, autonomic, and behavioral correlates of adaptation and maladaptation. Next, we present findings regarding human genetic polymorphisms in CRH pathway genes that are associated with stress and psychiatric disorders. Finally, we discuss a role for regulators of CRH activity as potential sites for therapeutic intervention aimed at treating maladaptive behaviors associated with stress.

## 1. Introduction

Corticotropin-releasing hormone (CRH) is a 41 amino acid peptide that was first isolated and characterized by Wylie Vale in 1981 [1]. It belongs to a family of peptides that has subsequently been found to include Urocortin1, 2, and 3 (Ucn1, 2, 3). This family of peptides shares 18% to 43% sequence homology, and its members are expressed in largely non-overlapping regions of the central nervous system and periphery. They function to maintain physiological activities ranging from appetite control to immune system modulation and mediation of the stress response [2]. 

### 1.1. CRH Synthesis

**Figure 1:**
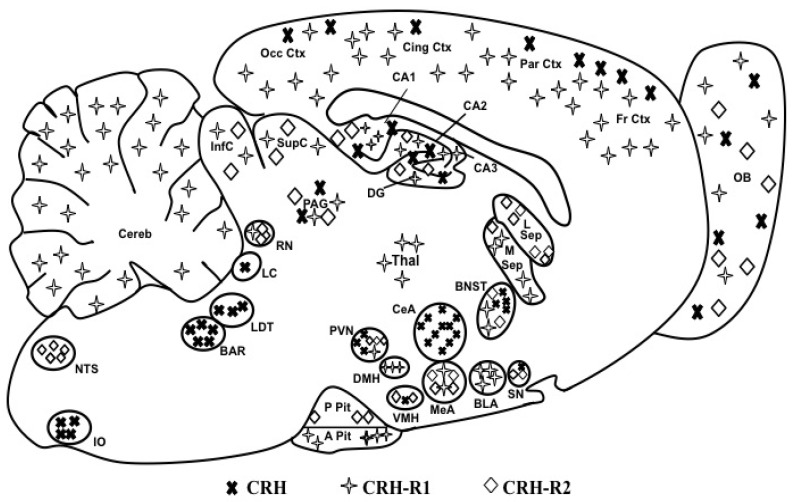
Expression of Corticotropin-releasing hormone (CRH), CRH-R1 and CRH-R2 mRNA in a normal mouse brain. CRH is synthesized in the PVN and shows high expression in the CeA, BnST and the hippocampus. CRH-R1 mRNA is highly expressed in the cortex, cerebellum, A Pit, hippocampus, BLA, MeA and the DMH. CRH-R2 displays high expression in the L Sep, SN, VMH, P Pit and the RN. Abundance of mRNA is shown as the density of representative symbols in an area. Abbreviations: Anterior pituitary, A Pit; Barrington’s nucleus, BAR; Basolateral nucleus of the amygdala, BLA; Bed nucleus of the stria terminalis, BnST; Central nucleus of the amygdala, CeA; Hippocampal areas, CA1, CA2, CA3; Cerebellum, Cereb; Cingulate cortex, Cing Ctx; Dentate gyrus, DG; Dorsomedial hypothalamus, DMH; Frontal cortex, Fr Ctx; Inferior colliculus, InfC; Inferior Olive, IO; Locus coeruleus, LC; Lateral dorsal tegmental nucleus, LDT; Medial nucleus of the amygdala, MeA; Medial septum, M Sep; Nucleus tractus solitarii, NTS; Occipital cortex, Occ Ctx; Olfactory bulb, OB; Posterior pituitary, P Pit; Periaqueductal gray, PAG; Parietal cortex, Par Ctx; Paraventricular hypothalamic nucleus, PVN; Raphe nucleus, RN; Supraoptic nucleus, SN; Superior colliculus, SupC; Thalamus, Thal; Ventromedial hypothalamus, VMH.

CRH is synthesized as a larger inactive preprohormone that undergoes proteolytic cleavage in the Golgi, mainly by prohormone convertase 2 (PC-2), and in some cases PC-1 [3,4] to generate mature active CRH. The major site of CRH synthesis is the parvocellular neurons of the paraventricular nucleus of the hypothalamus (PVN). Other brain regions that show high expression of CRH mRNA are the central nucleus of the amygdala (CeA), the bed nucleus of the stria terminalis (BnST), and other limbic areas including the hippocampus (Figure 1). In the PVN, CRH is packaged into secretory vesicles and released in a circadian rhythm or in response to stress. The packaged hormone is released from neurons in the PVN into the hypophyseal portal system that links the hypothalamus to the anterior pituitary gland. CRH binds to receptors on corticotroph cells in the anterior pituitary and stimulates the synthesis and secretion of the adrenocorticotropic hormone (ACTH), which ultimately activates adrenal glucocorticoid (GC) synthesis and secretion. These GCs (corticosterone in rodents, cortisol in humans) function to enable adaptation to the stressor and negatively feedback in the hypothalamus and pituitary (to suppress CRH and ACTH production) to establish homeostasis. In extra-hypothalamic parts of the brain, CRH acts in a neuromodulatory capacity on target neurons.

Arginine vasopressin (AVP) is a nonapeptide hormone involved in water homeostasis, HPA axis regulation, and behavioral and vascular tone. In HPA axis regulation, AVP is recognized as an important ACTH secretagogue that functions synergistically with CRH. As such, AVP has been shown to compensate for loss of basal, but not stress-activated, CRH-induced secretion of ACTH in CRH KO [5] and CRH-R1 KO mice [6,7]. Detailed AVP involvement in mediating HPA activity and stress-related behavior (see [8,9] for review) is beyond the scope of this review. However, it should be considered that in some studies described here in which HPA axis activity remains normal in the absence of CRH or CRH-R1, AVP may contribute to maintaining normal HPA axis activity.

### 1.2. CRH Receptors

CRH has two receptors, CRH-R1 and CRH-R2. These receptors share almost 71% amino acid sequence similarity [10], but are distinctly different in localization and affinities for CRH. In rodents, CRH-R1 displays widespread expression in the cortex and cerebellum, with higher expression in the anterior pituitary, hippocampus, basolateral and medial amygdala, dorsomedial hypothalamus, and parts of the pons/medulla and mesencephalon (Figure 1) [10,11]. CRH-R2, in contrast, is found in more discrete locations in the brain but displays abundant expression throughout the periphery. It is highly expressed in the lateral septum, supraoptic nucleus, ventromedial hypothalamus, cortical amygdala nucleus, raphe nucleus, choroid plexus, and the nucleus tractus solitarii (Figure 1). Regions of overlap between CRH-R1 and CRH-R2 include the olfactory bulb, hippocampus, superior and inferior colliculus, BnST, periaqueductal gray, and medial septum [10,11]. CRH-R1 has a much higher affinity for CRH than CRH-R2 and is the principle receptor involved in the hypothalamic-pituitary adrenal (HPA) axis mediated stress response. Ucn2 and 3 bind selectively to CRH-R2 [2], [12] while Ucn1 binds both receptors with high affinities [13]. 

In monkey brain, CRH-R1 immunoreactivity is most abundant in the pituitary, cerebellum, and brain stem, with moderate expression in the cerebral cortex, basal forebrain, basal ganglia and thalamus and weak expression in the prefrontal cortex and limbic areas [14]. CRH-R2 is present in the neocortex, pituitary, amygdala and hippocampus of monkey brain [15]. This is in sharp contrast to expression in the rat brain where only CRH-R1 is found in the pituitary and neocortex. 

Finally, in human brain, *CRHR1* is expressed in the hypothalamus, cerebellar cortex, pituitary, amygdala and nucleus accumbens [16] while *CRHR2* is expressed in the pituitary, amygdala, thalamus and hippocampus [17].

The first splice variants of CRH-R1 were identified as CRH-R1α and CRH-R1β [18]. CRH-R1α lacks 29 amino acids of exon 6 and is the highest affinity CRH receptor variant. CRH-R1β contains all 14 exons of the CRH-R1 gene, including exon 6, which decreases the affinity of CRH-R1β for CRH. Other CRH-R1 splice variants, including CRH-R1c-h, have been identified, all lacking exon 6 as well as other exons [19]. All the aforementioned CRH-R1 splice variants have been identified in humans, while only variants analogous to CRH-R1α, CRH-R1c, CRH-R1e, and CRH-R1f exist in mice. CRH-R2α and CRH-R2β splice variants have been identified in rodents with CRH-R2α expressed mainly in neuronal regions while CRH-R2β is found predominantly in peripheral regions such as the heart, blood vessels and duodenum [13,20,21]. In humans, both CRH-R2α and CRH-R2β are expressed in peripheral tissues and the brain. Both CRH-R1 and CRH-R2 receptors are G-protein coupled receptors (GPCR), composed of seven-transmembrane domains. Binding of CRH to either receptor results in a conformational change that produces activation of Gα_s_ stimulated adenylate cyclase and activated cyclic AMP and protein kinase A (PKA) signaling pathways [18,21]. The expression of different splice variants of CRH-R1 or CRH-R2 therefore mediate functions associated with their locations through activation of cAMP-mediated pathways.

The specific type of neurons that express the CRH receptors will also factor into their function. In that regard, a recent article by Refojo and colleagues identified the expression of mouse CRH-R1 in forebrain glutamatergic and GABAergic neurons, as well as midbrain dopaminergic neurons and to a lesser extent serotonergic neurons [22]. The functional effects of within each of these neuron populations will be discussed in later sections this review. 

### 1.3. CRH Binding Protein

The CRH binding protein (CRH-BP) is another source of regulation for CRH activity at the protein level. During the third trimester of pregnancy in humans, there is a significant increase in the release of placental CRH that predicts the relative timing for parturition [23]. Despite this dramatic release of CRH into circulation, there is no increase in ACTH secretion from the pituitary [24]. The most reasonable explanation for this came through the identification of the 37 kDa CRH-BP that sequesters CRH and prevents binding to its receptors [25]. In primates, CRH-BP is expressed in the brain, pituitary, liver, placenta and plasma [26] and has a higher affinity for CRH than CRH receptors do [27]. In rodents, CRH-BP is widely expressed in the cerebral cortex, lateral septum, olfactory bulb and limbic areas such as the CeA and the BnST as well as in pituitary corticotroph cells, with sparse expression in the PVN [27,28]. CRH-BP expression overlaps with CRH receptor expression and is therefore properly positioned to sequester CRH and inhibit its activity. Conversely, low levels of Ucns are also capable of binding CRH-BP and displacing CRH to increase levels of free CRH and thus, CRH activity [29]. Whether there are internal or external signals that allow for displacement of CRH by Ucns is unclear, but further investigation may provide insights into regulation.

### 1.4. Synaptic CRH Activity

A role for CRH in modulating CNS synaptic transmission outside the HPA axis has become evident. Extrahypothalamic CRH may act as a modulator of neurotransmitters, affecting their excitatory and inhibitory activity. CRH-R1 and CRH-R2 are metabotropic receptors and thus, function slowly in activating signaling cascades, unlike the fast activation of classical neurotransmitters, such as glutamate and GABA, on ionotropic receptors. Synaptic CRH would function to prime post-synaptic neurons for neurotransmitter or neuromodulator activity [30]. This activity of CRH is both receptor- and site-specific. For instance, CRH application depresses glutamate-mediated excitatory postsynaptic currents (EPSCs) in the CeA while facilitating these EPSCs in the lateral septum [31]. Application of CRH receptor antagonists has indicated that CRH-R1 is responsible for the changes in EPSCs in the CeA and lateral septum, while CRH-R2 facilitates EPSCs in the CeA and depresses them in the lateral septum, although Ucn1 mediates the latter. Further evidence in studies of mice lacking CRH-R1 in forebrain glutamatergic neurons shows that CRH increases excitatory field potentials in the BLA and facilitates action potential firing between hippocampal regions [22]. CRH modulation of synaptic activity is observed in other brain regions and provides evidence for the mechanism by which CRH mediates behavior. Evidence supporting this mechanism was demonstrated in a study where acute stress and CRH application facilitated long-term potentiation in the hippocampus and was responsible for enhanced contextual fear conditioning in mice [32]. Alterations in CRH expression and signaling are associated with a number of psychiatric disorders that are discussed later in this review. The interplay between extrahypothalamic CRH, its receptors and modulators may regulate synaptic activity that leads to the behavioral changes associated with these disorders.

## 2. CRH Agonists and Antagonists

A subpopulation of patients with major depression and post-traumatic stress disorder (PTSD) have elevated levels of CRH in their cerebrospinal fluid [33,34] Moreover, HPA axis hyperactivity is the most commonly observed neuroendocrine change in major depressive disorder (MDD). Substantial evidence suggests that normalization of the HPA axis might be a requirement for successful treatment of some individuals with MDD [35]. It should be noted that there is biological variation that occurs within these disorders and while some patients do exhibit CRH abnormalities, others do not [36,37]. Furthermore, the presence of CRH abnormalities may relate to the severity of the illness, or to the presence of melancholic features in patients with MDD [35]. The hyperactive CRH system and HPA axis abnormalities that occur in some patients suggests a potential role for CRH in modulating the neurocircuitry underlying mood and anxiety disorders. Animal studies have expanded the understanding of CRH activity in neuroendocrine and behavioral changes associated with stress. Important findings from these studies are discussed below.

The use of intracerebroventricular (ICV) CRH has provided further insight into CRH-mediated stress-induced behaviors. ICV injections of CRH in rats results in stimulation of hypothalamic and limbic brain regions that are activated in the stress response [38]. In animal models, several behavioral paradigms have been utilized that explore various aspects of anxiety, despair, fear, avoidance, and other stress-related behaviors. Paradigms such as the open field (OF), elevated plus maze (EPM), and light/dark preference (LD) tests are used as indices of anxiety. The forced swim (FST) and tail suspension (TST) tests are used to measure levels of despair, and fear conditioning is utilized as a measure of stress-induced learning and memory. These tests have been extensively validated and are used frequently in rodent studies. Some studies have demonstrated that ICV CRH increases locomotion, anxiety, fear, despair, and emotionality, decreases food intake, sexual activity, exploration [39] and social interaction and activates the HPA axis and the autonomic nervous system [40,41,42,43,44]. Furthermore, these changes can be reversed with CRH antagonists [45,46,47]. CRH-R1 antagonists and antisense oligonucleotides are anxiolytic in rats while CRH-R2 antagonists and antisense oligonucleotides increase despair-like behaviors and alter appetitive behaviors [48,49]. 

In vitro studies using rat amygdala and hypothalamic slices suggest that CRH and Ucn1 act through CRH-R1 to increase GABA release [50,51]. Moreover, prolonged daily infusion of Ucn1 into the BLA results in the development of anxiety-like behavior in the EPM and social interaction tests [52]. The study determined that this anxiety-like phenotype was inhibited with the administration of NMDA receptor antagonists. 

Ucn2 and Ucn3, which are primarily CRH-R2 ligands, have been demonstrated to suppress locomotor activity when injected into the intracerebral ventricles of rats [53,54]. The opposite effect occurs when ovine CRH is administered, it increases locomotor activity. Moreover, while CRH was anxiogenic in the EPM, Ucn3 was anxiolytic and Ucn2 had a delayed anxiolytic effect in the EPM test. These data are supported by evidence in CRH-R2 null mice (discussed in Section 3.3.2.) and reinforce the idea that CRH-R2 has a stress-coping function opposite to the stress-activating function of CRH-R1. In contrast to this data, Ucn2 administration in mice is anxiogenic in the EPM, an effect reversed by CRH-R2 specific antagonist [55]. Although much data supports an anxiolytic role for CRH-R2, there is still strong data supporting an anxiogenic role for the receptor. Despite the lack of consistency in data, CRH-R2 does have a role in mediating behavioral responses to stress. The degree of interaction with CRH-R1 in mediating these effects is yet to be determined. These studies and others also indicate the infusion of CRH or Ucn1, -2, or -3 in to the cerebral ventricle reduces feeding behavior in rodents [56]. 

Pharmacological manipulations have provided evidence in understanding central CRH system effects and continue to be important for investigating the effects of potential therapeutic drugs. In general, however, they are limited by the fact that their effects are short-lived and it is difficult to measure the degree of receptor activation mediating the observed effects. The generation of genetically altered animal models has allowed for a closer examination of the endogenous role for CRH. 

## 3. Genetically Altered Rodent Models

### 3.1. CRH Mutants

With the generation of CRH deficient mice (CRH KO) [57], the hypothetical role of CRH as an essential mediator of physiological responses to stress could be further tested. CRH KO mice from heterozygous matings are phenotypically normal compared to control mice [58]. Their only defining feature is a marked atrophy of the zona fasciculata of the adrenal gland and profound glucocorticoid deficiency. CRH KO mice generated from homozygous parents do not survive longer than 24 hours, due to a lack of glucocorticoids from the mother’s placenta to aid in proper lung formation. The aggregate studies in CRH KO mice have demonstrated that CRH KO mice display behavioral responses to stressors remarkably similar to those of wild-type (WT) animals [59,60]. These mice have low levels of basal plasma concentrations of corticosterone (CORT) that are not compensated for by other ACTH secretagogues such as vasopressin [58] and that do not increase with a foot shock stressor. Stress-induced activation of the HPA axis is absent in these mice whereas stress-induced behavioral responses thought to be mediated by CRH in the brain remain unaffected. It is of note that the use of two distinct CRH-R1 specific antagonists in WT and KO mice attenuated stress-induced behaviors. This indicates the existence of another CRH-like ligand, possibly one of the urocortins, acting through CRH-R1 [59].

### 3.2. CRH Overexpressing (CRH-OE) Mice

#### 3.2.1. General CRH-OE

To study the role of CRH hyperactivity, transgenic mice were generated that constitutively overexpress CRH (Table 1). Stenzel-Poore and colleagues [61] utilized the mouse metallothionein-I (MMT-1) promoter, expressed in the brain and peripheral areas such as the adrenal gland, heart, and testis, to drive CRH overexpression in these areas (CRH-Tg). Inserting rat CRH cDNA into Thy-1 regulatory genes created another model of CRH overexpression [62]. The Thy-1 gene drives neuronal expression postnatally through adulthood (CRH-OE_2122_). In addition, Lu *et al*. inserted a targeting allele consisting of a floxed stop codon, a CRH gene, and an IRES LacZ gene into the Rosa 26 allele (R26^+/flop CRH^) and mated them to Cre-lines to overexpress CRH [63]. The R26^+/flop CRH^ mice, when crossed with Deleter-Cre mice, overexpress CRH ubiquitously throughout the body (CRH-COE^Del^) [64]. These three general transgenic lines display elevated plasma CORT and, in some cases, elevated ACTH levels, altered HPA axis responses and Cushingoid phenotypes. Cushing’s syndrome is characterized by hypercortisolemia, truncal obesity, muscle wasting, thinning of the skin and hair loss [61]. These phenotypes are observed in all three transgenic lines described, although the CRH-OE_2122_ mice do not show a Cushing’s phenotype until six months of age [61,64,65].

Behavioral studies in CRH overexpressing mice show reduced locomotor activity in a novel environment that is further exacerbated by social defeat stress and increased anxiety identified by less time spent in the open arm of the EPM [64,66], and increased latency to enter the light compartment of a LD preference box [64,67] and black/white transition test [68]. ICV injection of α-helical CRH 9-41, a CRH antagonist, abolished this anxiogenic response [66]. Despite heightened anxiety, these mice displayed decreased despair, demonstrated by reduced immobility in the FST [64,67]. CRH-OE_2122_ mice have decreased acoustic startle reactivity, inability to habituate to a startle response, and a deficit in pre-pulse inhibition, all indicative of an impaired ability to process sensory information [62]. Moreover they have altered heart rate and increased food and water consumption [69]. The CRH-Tg mice from Stenzel-Poore and colleagues also show reduced alcohol preference [70]. This finding is surprising given that increased CRH-R1 activity is known to increase alcohol use in stressed rats and humans [71,72,73]. The data from these mice support a role for CRH in stress-mediated coping and sensory processing. These studies demonstrate that elevated CRH alters emotional regulation. However, analysis of the data may be confounded by the Cushing’s phenotype of these mice, including muscle wasting, which may alter their behavioral output. 

A recent article investigated the effects on GABA and glutamatergic transmission in CRH-OE_2122_ [74]. The study showed that constitutive overexpression of CRH reduced sensitivity to the anxiolytic effects of CRH-R1 antagonists, and GABA_A_ and glutamate receptor agonists in response to stress-induced hypothermia. Additionally, mRNA levels of distinct subunits of the GABA_A_ receptors and mGluR_2/3_ receptor were differentially altered in amygdala *versus* the hypothalamus [74]. The data supports the notion that an imbalance in GABAergic and glutamatergic transmission may underlie the genesis of stress-related maladaptive behaviors. 

#### 3.2.2. Spatially Restricted CRH-OE

The general overexpression of CRH in the aforesaid studies makes it difficult to identify which regions are involved in mediating the different phenotypes observed. This section focuses on transgenic mouse models that spatially restrict CRH overexpression to particular brain regions (Table 1). The Rosa 26 allele was inserted with a targeting allele consisting of a floxed stop codon, a CRH gene, and an IRES LacZ gene (R26^+/flop CRH^) [63]. These mice were bred to Nestin-Cre mice to overexpress CRH in neurons and glia from embryonic day 10.5 through adulthood (CRH-COE-Nes); Cam-Cre to overexpress CRH in forebrain glutamatergic neurons from postnatal day 15 (CRH-COE-Cam); and Dlx-Cre to overexpress CRH in GABA-ergic interneurons from embryonic day 10.5 into adulthood (CRH-COE-Dlx). None of these mutants display Cushingoid phenotypes and all exhibit normal basal CORT and ACTH levels. Stress-induced CORT and ACTH are however, higher in CRH-COE-Nes males compared to controls, an effect not observed in females. Behaviorally, CRH-COE-Nes mice show decreased despair behavior in both the FST and TST tests that is reversible with CRH-R1 antagonist treatment. The authors postulate that this is an adaptation to actively cope with stress [63]. The fact that these behaviors are only observed in the CRH-COE-Nes mice indicates that hindbrain regions are important for mediating the active coping responses observed in CRH-COE-Nes mice. Behavior in the CRH-COE-Cam mice indicates deficits in spatial memory in the Y-maze test and deficits in spatial learning in the Morris Water Maze (MWM) test [75]. These spatial learning and memory deficits recapitulate what is observed in mice that have undergone early life stress and do not occur in the absence of forebrain CRH-R1. This indicates that CRH-R1 signaling is important in mediating stress-induced alterations in hippocampus-dependent learning and memory. Further proof of this will be discussed in the CRH-R1 mutants’ section (Section 3.3.1.). A separate study also shows that overexpressing CRH in forebrain glutamatergic neurons (Crh-COE^CamCreERT2^) of mice increases anxiety in the EPM and LD tests, while anxiolysis is observed in the absence of forebrain CRH-R1 [22]. 

Sleep impairments are observed in a number of psychiatric disorders and are targets of therapy when treating patients with depression, PTSD, and anxiety disorders. CRH-COE-Nes and CRH-COE-Cam mice display increased REM (rapid eye movement) sleep, indicating a role for CRH dysregulation of sleep [76]. 

The R26^+/flop CRH^ mice have also been crossed to Pomc-Cre mice to overexpress CRH in the anterior and intermediate lobes of the pituitary (CRH-COE^APit^) [64]. CRH-COE^APit^ display a Cushingoid-like phenotype at 5–6 months old, decreased body weight but increased adrenal gland weight, increased basal CORT secretion that is arrhythmic, and no differences in the stress-induced CORT secretion, compared to controls, in males but a blunted effect in females. Behaviorally, these mice spent more time in the inner zone of the OF and less time immobile in the FST, indicative of decreased anxiety and despair-like behaviors, respectively [64].

#### 3.2.3. Spatially and Temporally Restricted CRH-OE

We have generated transgenic mice that overexpress CRH in the forebrain under the control of the CamKII promoter [77]. These mice utilize an inducible tetracycline system that enables CRH overexpression to be turned off in the presence of dietary doxycycline (FBCRHOE). CRH mRNA expression was found to be elevated in all forebrain regions excluding the thalamus and PVN. Lifelong forebrain CRH overexpression (FBCRHOE^life^) resulted in a Cushingoid-like phenotype and elevated nadir CORT and ACTH levels. This inducible tetracycline system was further used to study the importance of forebrain CRH overexpression during development in the first three weeks of life, E15 to P21 (FBCRHOE^dev^). Although FBCRHOE^dev^ mice have elevated basal CORT at P15 and P20, these levels normalize in adulthood. Behaviorally, FBCRHOE^dev ^mice display increased despair and anxiety-like behaviors as well as increased CRH-R1 mRNA that are reversed with antidepressant treatment [77]. A similar mouse model overexpressing forebrain CRH transiently from 8 to 11 weeks of age, shows increased cortex and hippocampal CRH and increased basal CORT levels with a trend towards mild anxiety in the LD test and active coping in the FST [78]. These studies demonstrate how disruption of CRH activity during critical developmental periods can affect behavioral outcomes. These studies have been invaluable in understanding the role of CRH in neuroendocrine and behavioral outcomes associated with stress.

### 3.3. CRH-R1 and CRH-R2 Mutants

CRH-R1 and CRH-R2 null mutant mice have been generated to elucidate the role for each receptor subtype in mediating the observed behaviors. The findings from these studies will be discussed in details below. 

#### 3.3.1. CRH-R1 Mutants

CRH-R1 null mutant mice were generated by deleting exons 5–8 of the CRH-R1 gene encoding the last 12 amino acids of the first extracellular domain through the fourth transmembrane domain and replacing them with a PGK neomycin-resistant gene cassette [79]. In behavior models used to measure anxiety, these mice showed reduced anxiogenic-like responses compared to the littermate controls [80]. A separate study generated CRH-R1 null mutant mice lacking the coding sequences of transmembrane regions V, VI, and VII, including the G-coupling protein domain and the intracellular cytoplasmic tail, resulting in a dysfunctional CRH-R1 unable to transmit any ligand-induced signals [81]. These mice displayed similar reductions in anxiety-related behaviors in response to ethanol withdrawal. To determine if CRH-R1 mediates anxiety-like behavior independently of the HPA axis function, Müller and colleagues generated conditional knockout mice with CaMKIIa driving Cre-mediated inactivation of CRH-R1 (Cam-CRHR1) in behaviorally relevant neuronal circuitries of the anterior forebrain and limbic system including the cortex, hippocampus and amygdala [82]. Similar to the conventional mutants, these conditional knockouts displayed significantly reduced anxiety-related behaviors indicating that selective disruption of the CRH-R1 signaling pathway reduces anxiety. Furthermore, diminished neuronal activity in regions such as the medial amygdala and the prelimbic cortex of these forebrian CRH-R1 KO mice likely mediates the anxiety phenotype observed [83]. Chronic social defeat stress or early life stress in WT mice causes cognitive impairments reflected in the Y-maze and MWM tests [75,84]. In Cam-CRHR1 mice that undergo the aforementioned stressors, loss of CRH-R1 in forebrain neurons protects against impairments in spatial performance. Moreover, these mice are protected from atrophy in the dendritic spines of CA1 and CA3 neurons and impaired hippocampal LTP. Interestingly, these CRH-R1 deficient mice appear to compensate for stress-induced impairments in synaptic transmission by increasing dendritic spines density and enhancing high-frequency stimulation LTPs in the hippocampus [75]. CRH activity through CRH-R1 receptors, therefore, not only mediates anxiety behaviors but functions in hippocampus-dependent cognitive performance. In contrast to forebrain CRH-R1 deletion, deletion of CRH-R1 in all brain neurons, using the Nestin promoter, decreases forced swim stress-induced alcohol consumption in adults [85] and increases basal and stress-induce plasma CORT in neonates [86]. Loss of CRH-R1 in all these transgenic lines results in differential HPA axis alterations that implicate divergent roles of forebrain and hindbrain regions in stress neuroendocrinology (Table 1) [6,79,81,86,87,88]. 

A recent study has also implicated divergent roles of CRH-R1 in different neurotransmitter systems. CRH-R1 was deleted specifically in forebrain glutamatergic neurons (Crhr1^Glu-CKO^) by crossing floxed CRH-R1 mice to Nex-Cre mice, Nex is a transcription factor that is expressed in mature glutamatergic neurons [22]. Crhr1^Glu-CKO^ mice display reduced anxiety in four distinct test, including EPM and LD preference tests. Conversely, mice with CRH-R1 deleted in midbrain dopamine neurons (Crhr1^DA-CKO^) display increased anxiety. These data indicate that different neurotransmitter system mediates opposite function of CRH-R1. While glutamatergic neurons mediate the anxiogenic properties of CRH-R1 activity, dopamanergic neurons mediate the anxiolytic functions. Further analysis of the Crhr1^DA-CKO^ mice showed a reduced prefrontal cortex response to stress-induced dopamine release. This study also demonstrated that CRH-R1 deletion in gabaergic and serotonergic neurons had no effect on anxiety-related behaviors. This data in general indicates that CRH may function in different neurotransmitter systems to balance CRH-R1 responses to stress [22]. 

#### 3.3.2. CRH-R2 Mutants

In contrast to the clear reduced-anxiety phenotype in both conventional and conditional CRH-R1 KO mice, significant differences in the behavioral phenotypes are reported amongst three independently generated CRH-R2 KO mouse lines. Subsequently, the physiological role of CRH-R2 in mediating anxiety is still unclear. CRH-R2 null mice have been demonstrated to increase HPA axis reactivity to stress [89]. Bale and colleagues generated a CRH-R2 null mutant mouse by constructing a targeting vector in which the portion of CRH-R2 encoding one-half of the fifth transmembrane domain through the end of the seventh transmembrane domain was deleted and replaced with a neomycin-resistant gene cassette [90]. In contrast to CRH-R1 mutants, behavioral studies revealed that these mice displayed increased anxiety-like behaviors. Similar increased anxiety and despair-related behaviors have been found in separate studies of CRH-R2 null mice [91,92]. CRH-R2 null mutants have also been produced by homologous recombination with a targeting construct containing CRH-R2 and a neomycin cassette [91]. Behavioral studies revealed no difference in anxiety-like behaviors in these mutants. Compared to control mice, these CRH-R2 null mutant mice were also tested for despair-like behavior using the FST and showed longer immobility time indicating increased despair-like behaviors [93].

When reared in an isolated environment after weaning, CRH-R2 deficient mice displayed increased locomotor behavior to a greater extent than what is observed in WT mice [94]. 

#### 3.3.3. Combined CRH-R1 and CRH-R2 Mutants

Mice null for both CRH-R1 and CRH-R2 display adrenal cortex atrophy, increased PVN CRH, decreased basal CORT and ACTH levels and diminished HPA axis reactivity in response to stress [90]. A similar phenotype is observed in CRH-R1 deficiency alone, indicating that CRH-R2 cannot overcome the loss of CRH-R1 [7]. A separate study observed that CORT and ACTH levels were significantly different between CRH-R1/CRH-R2 double KO compared to CRH-R1 KO but both were significantly diminished compared to controls. Further examination indicated that, CRH-R1 KO male mice born from dams who were either CRH-R2 het or CRH-R2 KO, show increased anxiety in OF and EPM, implying that there are influences of genetic background on behavior. Female mice showed decreased anxiety in the EPM, also indicating gender dimorphism and perhaps the influence of gender-specific hormones in mediating behavior [95].

#### 3.3.4. Urocortin Mutants

Urocortins (Ucn) have a high affinity for CRH receptors. While Ucn1 and CRH have high affinities for CRH-R1, Ucn2 and 3 are considered to be the major endogenous ligands for CRH-R2 [96]. CRH deficient mice do not differ from WT mice in their freezing response to a foot shock stressor, but CRH-R1 antagonists block freezing behavior in CRH-KO mice [59]. This brought about the idea that another CRH-related peptide was compensating for loss of CRH. The most likely candidate was Ucn1 since it also has a high affinity for CRH-R1. Ucn1 null mice however, have normal HPA axis activity and anxiety phenotypes, but display impairments in acoustic startle response, not due to hearing impairments [97]. Studying the response of mice deficient for both CRH and Ucn1 to foot shock may better elucidate whether Ucn1 acts on CRH-R1 in the absence of CRH. A different Ucn1 null mouse line generated by a different group shows that Ucn1 deficiency increases anxiety in the OF and EPM [98]. However, more data on this line of mice is needed as the hearing impairment and reduction in CRH-R2 mRNA in the lateral septum in these mice may account for the increased anxiety. These Ucn^-/-^ mice also have reduced ethanol preference [99], implicating a role for Ucn1 in alcohol behavior. 

In contrast to Ucn1 null mice, Ucn2 null mice have altered HPA axis regulation of CORT and ACTH [100]. Ucn2 KO female mice show higher nocturnal CORT and ACTH levels, not observed if the mice were ovariectomized, implicating estrogen mediation of this effect. The female Ucn2 KO mice also have an increase in hypothalamic AVP mRNA that may account for the rise in ACTH and CORT. Behaviorally, Ucn2 KO mice show no anxiety phenotypes but the females do display less despair in the FST and TST tests [100]. This implicates that Ucn2 may be involved in mediating gender differences in relation to HPA axis function and depressive phenotype.

When both Ucn1 and 2 are deleted (Ucn1/Ucn2 dKO), there is a stress-induced elevation in CORT (males only) as well as elevated CRH mRNA in the PVN [101]. Ucn1/Ucn2 dKO mice display reduced basal anxiety as well as reduced stress-induced anxiety in the OF and LD tests. Since Ucn1 null mice do not show changes in HPA axis regulation, it is plausible that an interaction between Ucn1 and 2 activities is necessary to influence HPA axis response. 

Ucn3 deficient mice (Ucn3^tZ/tZ^) only display behavioral alterations in the social discrimination task [102]. Whereas controls show no discrimination of a familiar versus unfamiliar subject, Ucn3^tZ/tZ^ are able to discriminate. This was also the case for CRH-R2 KO mice but not Ucn2^tZ/tZ^, indicating that this behavior is specific to Ucn3 action on CRH-R2 receptors. Ucn3^tZ/tZ^ demonstrate no alterations in HPA axis activity or in tests of anxiety, despair, novel object recognition, and conditioned fear. 

When all three Ucn are deleted, the triple knock out (tKO) exhibit increased anxiety in the OF and LD tests 24 hours after stress but are no different from WT mice in unstressed conditions or immediately after stress [103]. The tKO mice display an increase in the acoustic startle response 24 hours post-stress. They also increase freezing in cued fear conditioning but not the contextual test, and have stress-induced deficits in spatial learning in the MWM test. Amygdala gene expression profiles demonstrate that Ucn tKO mice do not have typical changes in stress-induced genes to aid in coping with stress. Thus urocortins appear to be essential for recovery after stress, by modifying expression of stress-related genes in the amygdala. This tKO line may be useful in studying the proposed function of CRH-R2 in reducing sensitivity to stress [21]. 

Due to the discrepancy in two lines of Ucn1 null mice, the function of Ucn1 in anxiety remains unclear. However, it is clear that Ucn1 is not important for mediating HPA axis activity, a role more attributable to Ucn2 function. The data discussed here also point to a role for Ucn2 activity in mediating despair behavior and stimulating gender differences. The specificity of Ucn3^tZ/tZ^ mice in affecting only social discrimination indicates a role for Ucn3 in processing social cues. This role of Ucn3 is fitting as it is expressed in regions associated with the accessory olfactory system. 

### 3.4. CRH-BP Mutants

Mice overexpressing CRH-BP were created by inserting a fragment of the rat CRH-BP, between the mouse metallothionein-I promoter and a 2.1 kb noncoding fragment of the human growth hormone, which contains a polyadenylation sequence. These mice overexpress CRH-BP in the brain and pituitary as well as peripheral sites such as the placenta, plasma, and amniotic fluid [27]. Male transgenic mice showed increased weight gain, to a greater extent than females, and no HPA activity changes [104]. Overexpressing CRH-BP only in the anterior pituitary, using the pituitary glycoprotein hormone promoter, similarly produced normal circadian and stress-induced levels of ACTH and CORT [105]. Further investigation demonstrated compensatory increases in both PVN CRH and vasopressin (AVP) as the culprits for lack of HPA axis alterations. The behavioral phenotype of these mice includes increased locomotion and a trend towards decreased anxiety in the OF [105].

Interestingly, CRH-BP deficient mice likewise display normal circadian and stress-induced levels of ACTH and CORT. However, anxiety phenotypes are much more evident in these mice in the EPM, OF and defensive withdraw tests, with females exhibiting more anxiety [106]. CRH-BP deficient male mice demonstrate reduced food intake and diminished weight gain, reflecting the defined role for CRH in anorexiogenic behavior. The fact that all these mutant CRH-BP mice display normal HPA axis activity (Table 1) underscores the importance of maintaining homeostasis in this pathway, conceivably, through compensatory changes in PVN CRH and AVP transcript levels.

**Table 1:** Genetically Altered Rodent Models.

CRH Deletion Mutant			
Line	Manipulation	Main Phenotypes	References
CRH- KO	Constitutive deletion of CRH by insertion of a phosphoglycerate kinase neomycin-resistant cassette	Adrenal insufficiency	[57,58,59,60]
		↓ Stress CORT	
		No behavioral changes	
**CRH Overexpression (OE) Mutants**			
**Line**	**Manipulation**	**Main Phenotypes**	**References**
CRH-Tg	Mouse metallothionein-1 (MT-1) promoter driven CRH OE in brain, adrenal glands, heart, and testes.	Adrenal Hypertrophy	[61,66,67,70,79,80]
		Cushingoid phenotype	
		Attentional Impairment	
		↑ Basal CORT and ACTH	
		↓ Locomotion	
		↑ Anxiety in OF, EPM, LD, and black/white transition test	
		↑ Active coping in FST	
		↓ Despair in FST	
		↓ Sexual receptivity in females	
		↓ Alcohol preference	
		Gene expression changes	
CRH-OE_2122_	Thy-1 promoter driven CRH OE in neurons postnatally through adulthood.	Adrenal Hypertrophy	[62,65,69]
		Cushingoid phenotype at 6 months of age	
		↑ Basal CORT	
		Dexamethasone non-suppression	
		↓ Acoustic startle reactivity	
		↓ Habituation to a startle response	
		Deficit in pre-pulse inhibition	
		↑ Food and water consumption, and altered heart rate	
CRH-COE^Del^	Rosa26 (R26) promoter driven CRH OE in the whole body.	Cushingoid phenotype at 3-weeks of age	[64]
		↑ Adrenal weight, ↓ thymus weight	
		↑ Basal CORT	
		↑ Anxiety in OF, EPM, LD, and black/white transition test	
		↓ Despair in FST	
**CRH Overexpression (OE) Mutants**			
**Line**	**Manipulation**	**Main Phenotypes**	**References**
CRH-COE^APit^	R26 and POMC promoter driven CRH OE in the anterior and intermediate lobes of the pituitary	Mild Cushingoid phenotype at 5-6 months of age	[64]
		↑ Basal CORT	
CRH-COE-Nes	R26 and Nestin promoter driven CRH OE in neurons and glia from embryonic day 10.5	↑ Stress-induced CORT and ACTH in male mice	[63,75,76]
		↓ Despair in FST and TST tests, reversible with CRH-R1 antagonist treatment	
		↑ REM sleep	
CRH-COE-Cam	R26 and CamK2 promoter driven CRH OE in forebrain glutamatergic neurons from postnatal day 15	Normal HPA axis activity	
		↑ REM sleep	
		↑ Deficit in spatial performance in the MWM and Y-maze tests.	
CRH-COE-Dlx	R26 and Dlx promoter driven CRH OE in GABAergic interneurons from embryonic day 10.5	Normal HPA axis activity and behavior	
FBCRHOElife	CamK2 promoter driven forebrain CRH OE from embryonic day 0 through life	Cushingoid phenotype by 8 weeks of age	[77]
		↑ Nadir CORT and ACTH	
Crh-COE^CamCreERT2^	R26 and Camk2a-CreERT2 promoter driven CRH OE in forebrain glutamatergic neurons (OE induced by tamoxifen at postnatal week 8)	↑ Anxiety in LD and EPM tests	[22]
FBCRHOEdev	CamK2 promoter driven forebrain CRH OE from embryonic day 15 to postnatal day 21	↑Basal CORT only during CRH- OE.	[77]
		↑ Despair in FST and TST test (↓ despair in FST with antidepressants treatment)	
		↑ Anxiety in OF, EPM, and LD tests	
		↑ CRH-R1 mRNA in the cingulate cortex, dentate gyrus and CA1 region of the hippocampus	
CRF-OE	CamK2 promoter driven forebrain CRH OE from 8 to 11 weeks of age	↑ Nadir CORT	[78]
		↓ Thymus weight in females	
		↓ Locomotion in familiar environment	
		↓ Despair in FST	
		Trend towards anxiety in LD test	
**CRH Receptor Mutants**			
**Line**	**Manipulation**	**Main Phenotypes**	**References**
CRH-R1^-/-^	Constitutive deletion (exons 5-8) of the CRH-R1	Adrenal gland atrophy	[79,80]
		↑ PVN CRH	
		↓ Basal CORT	
		↓ Stress-induced CORT and ACTH in males	
		↓ Anxiety in LD and EPM tests	
CRH-R1^-/-^	Constitutive deletion of CRH-R1 (transmembrane regions V, VI, and VII)	↓ CRH-induced cAMP	[6,81,83,87,88]
		↓ Basal ACTH in pituitary cultures	
		↓ Basal CORT in females	
		↑ PVN CRH only in neonates	
		↑ Plasma and PVN vasopressin	
		↓ Stress-induced CORT and ACTH	
		↓ ACTH-induced CORT	
		↓ Anxiety in LD test basally and during alcohol withdrawal	
		↓ Neuronal activity (cFOS)	
Crhr1^loxP/loxP^Camk2a-cre Or Cam-CRHR1 Or CRHR1^Camk2aCre^ Or CRF_1_-CKO	Camk2a promoter driven deletion CRH-R1 in forebrain	↑ Stress-induced CORT and ACTH in adults and neonates	[75,82,84,86]
		↑ CRH mRNA in PVN in adults and neonates	
		↓ Anxiety in LD and EPM test in adults	
		Cam-CRHR1	
		Hyperactive in OF test in adults	
		↓ Neuronal activity	
		↓ Deficit in spatial memory in Y-maze tests after chronic social defeat stress	
		No deficit in spatial learning and memory in MWM or Y-maze tests after early life stress	
		No chronic social defeat stress-induced deficits in the novel object recognition test	
		No stress-induced atrophy of apical dendrites in CA3 neurons compared to control mice	
		No stress-induced reduction of GR mRNA in CA1 and CA3 neurons compared to control mice	
		↑ Weight gain after chronic stress	
		No deficit in hippocampal LTP after early life stress	
		↑ High frequency stimulation-induced LTP with early life stress	
Crhr1^loxP/loxP^Nes-cre Nes-CRHR1	Nes-Cre promoter driven deletion CRH-R1 in all neurons	↓ Basal CORT in neonates	[85,86]
↑ Stress-induced CORT and ACTH			
↓ Alcohol consumption after forced swim stress			
**CRH Receptor Mutants**			
**Line**	**Manipulation**	**Main Phenotypes**	**References**
Crhr1^Glu-CKO^	Nex-Cre promoter driven CRH-R1 deletion in mature glutamatergic neurons	↓ Anxiety in LD, EPM, novel object exploration, and modified hole board tests	[22]
		No effect on despair behavior in FST	
		No effect auditory fear conditioning	
		↑ Locomotion in LD test	
		No effect on basal or stress-induced CORT secretion	
		↓ Excitatory field potentials on glutamatergic neurons in the BLA	
		↓ Facilitation of action potential firing between hippocampal DG-CA3-CA1 network	
Crhr1^DA-CKO^	Dat-CreERT2 promoter driven CRH-R1 deletion in midbrain dopaminergic neurons	↑ Anxiety in LD, EPM, novel object exploration, and modified hole board tests	
		No effect on despair behavior in FST	
		No effect auditory fear conditioning	
		No effect on basal or stress-induced CORT secretion	
		↓ Response to stress-induced DA release in PFC	
Crhr1^GABA-CKO^	DLX5/6 promoter driven CRH-R1 deletion in forebrain GABAergic neurons	No effect on anxiety behaviors	[22]
		No effect on despair behavior in FST	
Crhr1^5HT-CKO^	ePet-Cre promoter driven CRH-R1 deletion in brainstem seratonergic neurons	No effect auditory fear conditioning	
		No effect on basal or stress-induced CORT secretion	
Crhr1^CNS-CKO^	Nes-Cre promoter driven CRH-R1 deletion in all neurons	No effect on anxiety behaviors	
CRH-R2^-/-^	Constitutive deletion by replacing 5th-7th transmembrane domains with a neomycin-resistant cassette	↑ Stress-induced CORT and ACTH	[90,93]
		↓ Food intake after stress of food deprivation	
		↑Anxiety in EPM and OF tests	
		↑ Despair in FST	
		↑ CRH mRNA in the CeA	
		↑ Ucn1 mRNA in Edinger-Westphal (EW) nucleus	
CRH-R2^-/-^	Constitutive deletion by replacing exons of the 3rd intracellular loop with neomycin-resistant cassette	↑ Anxiety in EPM and LD tests in males	[91,92]
		↑ Locomotion in OF test in males	
		↑ Stress-induced anxiety in the OF test in males	
		↓ Neuronal activation measured by levels of phosphorylated CREB	
		↑ Despair behavior in the FST and TST tests that is prevented when MEK/ERK pathway in the hippocampus is inhibited	
CRH-R2^-/-^	Constitutive deletion by replacing the 3rd and 4th transmembrane domains with a neomycin-resistant cassette	↓ cAMP activity in cultured cardiomyocytes	[89]
		↑ Ucn1 mRNA in Edinger-Westphal (EW) nucleus	
		↑ Stress-induced CORT and ACTH	
		↓ Cardiac function with Ucn administration	
		Altered feeding with Ucn administration	
CRH-R1^-/-^/	CRH-R1^-/-^ [79] were crossed to CRH-R2^-/-^ [90] to generate these double knockout mice	↑ PVN CRH	[7]
CRH-R2^-/-^		↓ Basal CORT and ACTH	
		↓ HPA axis reactivity to stress	
CRH-R1^-/-^/	CRH-R1^-/-^[81] were crossed to CRH-R2^-/-^[89] to generate these double knockout mice	↓ Basal CORT and ACTH	[95]
CRH-R2^-/-^		↓ Anxiety in EPM and OF tests in females	
		↑ PVN CRH and AVP	
**Ucn mutants**			
**Line**	**Manipulation**	**Main Phenotypes**	**References**
Ucn^-/-^	Constitutive deletion of Ucn1 by replacement of the coding exon of Ucn with an EGFP-LacZ fusion reporter and a *PGKneo* selection cassette	Normal HPA axis activity	[97]
		No change anxiety in the EPM, OF, and LD tests	
		Impaired acoustic startle response	
Ucn^-/-^	Constitutive deletion of Ucn1 by replacement of region encoding the mature peptide with a neomycin-resistant gene cassette	Normal HPA axis activity and feeding behavior	[98,99]
		↑ Anxiety in EPM and OF tests	
		↓ CRH-R2 mRNA in the LS	
		↓ Length of hair cell in organ of Corti leading to hearing impairments	
Ucn2 KO	Constitutive deletion of Ucn2 by insertion of a neomycin-resistant gene cassette	↑ Nocturnal CORT and ACTH in females	[100]
		↑ AVP mRNA in the PVN and SON of females - altered drinking habits	
		↓ Despair in the FST and TST in females	
		No changes in anxiety in EPM and LD tests or in conditioned fear tests	
		↑ CRH mRNA in the BnST and CeA	
		↓ Ucn3 mRNA in the median preoptic nucleus and perifornical area	
		↑ CRH-R2 mRNA in the BnST, LS and DR	
Ucn1/Ucn2 dKO	Cross breeding Ucn1 [98] and Ucn2 [100] single KOs to generate these double knockout mice.	↑ Stress-induced plasma CORT in males	[101]
		↑ PVN CRH mRNA	
		Hypertrophy of the zona fasciculate	
		↓ Anxiety in EPM and OF	
		↓ Behavioral response to acute stress in females	
		↓ CRH-R2 mRNA in the LS	
		↑ Amygdala CRH mRNA	
Ucn3^tZ/tZ^	Ucn3 gene was disrupted by homologous recombination and the ORF was replaced by a tau-lacZ reporter gene	Normal basal and stress-induced HPA axis responses	[102]
		No changes in anxiety-related behaviors in EPM, social interaction, and modified hole board tests compared to WT	
		No difference in despair behavior in the FST compared to WT	
		No genotype effect in the ASR test	
		↑ Cocial discrimination memory	
Ucn tKO	Cross breeding Ucn1, 2, and 3 single KOs from [Vetter, Chen, deussing]	↓ Basal exploration in OF 24 hours post-stress	[103]
		↑ Anxiety in OF, LD and ASR tests 24 hours after an acute stressor	
		↑ Freezing in cued fear conditioning and ASR tests	
		↓ Spatial learning in MWM	
		↑ CRH-R2 mRNA in the LS and DRN	
		↑ CRH-R1 mRNA in amygdala compared to controls 24 hours post- stress	
		Lack of stress-induced amygdala gene modification compared to that observed in controls	
**CRH-BP mutants**			
**Line**	**Manipulation**	**Main Phenotypes**	**References**
CRH-BP (transgenic)	Mouse metallothionein-1 (MT-1) promoter driven CRH-BP OE in the brain and pituitary as well as sites such as the placenta, plasma, and amniotic fluid	Normal CORT and ACTH	[104]
		↑ Weight gain (gender specific)	
		Blunted ACTH response to LPS injection	
CRH-BP (transgenic)	Pituitary glycoprotein hormone a-subunit (a-GSU) promoter driven CRH-BP OE in the pituitary	Normal CORT and ACTH	[105]
		↑ PVN CRH and vasopressin	
		↑ Locomotion	
		Trend towards decreased anxiety in the OF	
CRH-BP^-/-^	Constitutive deletion by replacing exons1-5 with a phosphoglycerate kinase neomycin-resistant cassette	Normal CORT and ACTH	[106]
		↑ Anxiety in EPM, OF and defensive withdraw (gender specific)	
		↓ Food intake and weight gain in males	

(Abbreviations: Acoustic startle response test, ASR; Adrenocorticotropin Releasing Hormone, ACTH; Arginine Vasopressin, AVP; Basolateral nucleus of the amygdala, BLA; Bed nucleus of the stria terminalis, BnST; Central nucleus of the amygdala, CeA; Corticosterone, CORT; cAMP response element-binding, CREB; Corticotropin-Releasing Hormone, CRH; Dentate gyrus, DG; Dopamine, DA; Dorsal raphe nucleus, DRN; Elevated Plus Maze, EPM; Forced Swim Test, FST; Lateral Septum, LS; Light/Dark Preference test, LD; Lipopolysaccharide, LPS; Long-term potentiation, LTP; Morris Water Maze, MWM; Open field test, OF; Open Reading Frame, ORF; Overexpression, OE; Paraventricular nucleus of the hypothalamus, PVN; Prefrontal Cortex, PFC; Proopiomelanocotin, POMC; Rapid Eye Movement, REM; Supraoptic nucleus, SON; Tail suspension test, TST; Urocortin, Ucn).

### 3.5. The Use of Viral Vectors to Modulate CRH Activity

In the bulk of studies thus far described, the relatively broad brain regions investigated constrain our ability to understand the role of CRH action in restricted brain nuclei in mediating behaviors. The use of lentiviral vectors has succeeded at overcoming this limitation by allowing for precise spatial regulation of CRH overexpression (Table 2). Regev *et al*. (2010) injected a lentiviral vector, containing rat CRH cDNA, into the CeA of 7-week old male mice. The authors presented that chronic (4-month) overexpression of CRH in the CeA of male mice under basal conditions had minor effects on anxiety in the OF and LD preference tests [107]. However, 30 minutes of restraint stress significantly attenuated anxiety-related behaviors, implying possible habituation to a stressor. In a separate study, short-term inducible CeA CRH overexpression increased anxiety after stress [108]. Since CeA CRH is known to be anxiogenic, perhaps the 4-month long CeA CRH OE results in compensatory changes that cause the observed habituation response. In male rats, extended elevated CeA CRH (10 weeks) during adulthood differentially dysregulated HPA axis function and increased CRH in the PVN [109]. Observed behaviors included decreased locomotion in the OF test, increased anxiety in EPM, and increased time withdrawing and under protection in the defensive withdrawal test. A similar study elevating CeA CRH levels in female rats demonstrated stress pathology changes including increased anxiety and despair-like behaviors, and impaired negative feedback of the HPA axis [110]. These female rats show augmented basal anxiety in the acoustic startle response. It is notable that the lentiviral construct used in this latter study was generated to infect neurons that endogenously produce CRH. This may limit confounds of expressing CRH in neurons that do not normally express CRH, and subsequent effects of these neurons on their targets. It therefore appears that short term CeA CRH OE results in anxiogenic phenotypes while longer term CeA CRH OE causes a blunting of anxiety behaviors. We can however not exclude the effects of gender and/or species differences in mediating the discrepancies in these studies.

Like the CeA, the BnST is a part of the extended amygdala involved in anxiety and fear mediation. Two studies of CRH-OE in the BnST in rodents, using distinct lentiviral constructs, show no effect on anxiety behavior in the EPM and defensive withdrawal tests [107,111]. Both studies also demonstrate no HPA axis alterations and compensatory reductions in CRH-R1 mRNA in the BnST. Fear potentiated-startle (FPS) is a behavioral paradigm in which acoustic noise (unconditioned stimulus, US) is paired with a foot-shock (conditioned stimulus, CS). Measures of startle response and shock reactivity in response to only the US provide information on acquisition, retention, and expression of fear learning. CRH-OE in the BnST of male rats prior to fear conditioning impairs acquisition of fear, as these rats have attenuated startle and shock reactivity amplitudes [111]. However if CRH is overexpressed after fear conditioning, the BnST CRH-OE mice have potentiate fear responses compared to controls, indicating enhanced retention or expression of fear memory. This abnormal enhancement of FPS is observed in individuals with PTSD, and points a potential mechanism in which elevated BnST CRH activity strengthens association of neutral cued with fear memories.

Environmental enrichment (EE), which is known to reduce anxiety, significantly decreases CRH-R1 mRNA in the BLA [112]. Lentiviruses containing small interference RNA (siRNA) were generated to knock down CRH-R1 in the BLA to further investigate its role in anxiety. Behavioral data indicates that siRNA knockdown of BLA CRH-R1 decreased anxiety in the OF and LD and a trend in EPM. The authors suggest the BLA as a potential target of therapeutics that mimics the anxiolytic effects of EE and benzodiazepines [112]. Comparatively, when this approach was used to knockdown CeA CRH, mice show decreased anxiety in the EPM [108]. Acute restraint stress, which was anxiogenic for control mice, had no effect on CeA CRH-KD mice. Since restraint stress is known to increase CeA CRH and cause anxiety, knockdown of CeA CRH by the viral vector may counteract the effects of stress. This data indicates that CRH-R1 in both the BLA and CeA functions to mediate anxiety responses.

Limbic regions are not the only modulators of CRH activity in anxiety behavior. This is demonstrated in a study that used lentiral-based siRNA to knock down 60% of CRH-R1 receptors in the external part of the globus pallidus of the striatum (GPe) [113]. This increased anxiety in LD, OF and cause a trend towards increased anxiety in the EPM. CRH-R1 antagonist infused into the GPe also increases anxiety in OF and marble burying tests. These data imply that, in contrast to anxiogenic effects of CRH-R1 activation in the amygdala, activation in the GPe has anxiolytic properties. In this study, restraint stress reduced CRH-R1 mRNA in GPe, further verifying the anxiolytic properties of GPe [113].

**Table 2:** Viral Vectors in Rodent Models.

Line (viral vector)	Manipulation	Main Phenotypes	References
CeA CRF OE	Long-term lentiviral CRH OE in the CeA of adult male mice.	↓ Basal and stress-induced anxiety in OF and LD tests	[107]
(pCSC-SP-PW-rCRF-IRES/GFP)	Behavioral testing began 4 months after lentiviral injections		
		↓ Response to acoustic startle	
		Habituation to startle after stress ↑ CRH-R1 mRNA in CeA	
CeA-CRF-OE	Short-term lentiviral CRH OE in the CeA of adult male mice when Dox is administered.	↑ Stress-induced anxiety in LD test	[108]
(rtTA-IRES/GFP + TRE-mCRF-IRES/RFP)			
	Behavioral testing began 3 days after Dox administration	No effects on despair in FST or TST	
		No effects on fear conditioning	
Lenti-CMV-CRF OE	Lentivirus-induced CRH OE in the CeA of female rats.	Impaired negative feedback of the HPA axis	[110]
(LVCRFp3.CRF)	Behavioral testing began 2 weeks after lentiviral injections		
		Disrupted reproductive and sexual function.	
		↑ Despair in FST	
		↑ Anxiety in acoustic startle	
CeA CRF OE	Lentivirus-induced CRH OE in the CeA of male rats.	↑ CRH and vasopressin mRNA in the PVN	[109]
LVCRFp3.CRF	Behavioral testing began 4 weeks after lentiviral injections		
		↑Basal ACTH	
		Dexamethasone non-suppression	
		↑ Anxiety in EPM and defensive withdrawal tests.	
BnST CRF OE	Long-term lentiviral CRH OE in the BnST of male mice.Behavioral testing began 4 months after lentiviral injections	↑ Despair in FST	[107]
(pCSC-SP-PW-rCRF-IRES/GFP)		↓ CRH-R1 mRNA in BnST	
BnST CRF OE	CRH OE in the BnST of adult male rats before or after fear conditioning in ASR tests	No changes in anxiety measures in EPM or DW tests	[111]
(LVCRFp3.CRF)			
		No HPA axis alterations	
	Behavioral testing began ~2 weeks after lentiviral injections	↓ CRH-R1 binding density in the BnST	
		↓ CRH-R2 binding density in the DRN	
		CRH OE induced before conditioning to fearful stimulus:	
		No differences in baseline ASR	
		↓ Startle sensitization and shock reactivity in ASR	
		↓ FPS, impaired acquisition of associative fear memory	
		CRH OE induced after conditioning to fearful stimulus:	
		↑ FPS, enhanced fear memory expression	
BLA CRFR1 KD	CRH-R1 KD in the BLA of adult male mice.	↓ Anxiety in the LD and OF tests	[112]
(Lenti-shCRFR1)	Behavioral testing began ~2 weeks after lentiviral injections		
CeA CRF-KD	CRH KD in the CeA of adult male mice	↓ Basal anxiety in the EPM test and stress-induced anxiety in LD test	[108]
(Lenti-shCRF)			
	Behavioral testing began ~2 weeks after lentiviral		
		No effects on despair in FST or TST	
		No effects on fear conditioning	
		↑ Basal plasma CORT levels	
		↓ Ucn3 mRNA in BnST	
GPe CRFR1 KD	CRH-R1 KD in the GPe of adult male mice.	↑ Anxiety in the LD, OF and EPM tests	[113]
(Lenti-shCRFR1)	Behavioral testing began ~2 weeks after lentiviral injection		
		No changes in locomotion	
		↓ Enkephalin protein in GPe, possible mechanism for increased anxiety	
rPFA-Ucn3 OE	Transgenic mice with Ucn3 under the control of a TRE were injected in the rPFA with lentivirus containing rtTA. Ucn3 OE occurs when Dox is administered	↑ Anxiety in the LD and OF tests	[114]
(Lenti-rtTA)		↑ Metabolic rate, but has no effect on food intake	
		↓ Insulin sensitivity	

(Abbreviations: Adrenocorticotropin Releasing Hormone, ACTH; Acoustic Startle response test, ASR; Bed nucleus of the stria terminalis, BnST; Basolateral nucleus of the amygdala, BLA; Central nucleus of the amygdala, CeA; Corticosterone, CORT; Corticotropin-Releasing Hormone, CRH; Corticotropin-Releasing Factor, CRF; Dorsal Raphe Nucleus, DRN; Doxycycline, Dox; Elevated Plus Maze, EPM; Fear Potentiated Startle, FPS; Forced Swim Test, FST; Globus Pallidus (external), GPe; Hypothalamic-Pituitary-Adrenal, HPA; Knock-down, KD; Dark/Light Preference test, LD; Overexpression, OE; Open field test, OF; Perifornical Area (rostral), rPFA; Tail suspension test, TST; Tetracycline Response Element, TRE; Urocortin, Ucn).

Lentiviral vectors have also been used to overexpress Ucn3 in the rostral perifornical area (rPFA) of the hypothalamus that projects to neurons in the lateral septum and ventromedial hypothalamus [114]. Mice overexpressing Ucn3 in the rPFA display increased anxiety, which is most likely through rPFA Ucn3 acting on CRH-R2 neurons in the lateral septum. 

The use of lentiviral vectors also produced the added advantages of infection neurons and activating CRH receptors on neurons that they project to, unlike pharmacological methods that only act locally. Although the use of viral vectors is still ectopic, it provides more physiological relevance that pharmaceutical methods and more spatial relevance than genetically altered rodents alone.

## 4. Human Gene Polymorphisms

CRH hyperactivity is present in several psychiatric disorders. For example, CRH levels are elevated in the CSF of some post-traumatic stress disorder (PTSD) and depressed patients [33,34]. Furthermore, this HPA axis dysregulation, which occurs in a subpopulation of individuals who suffer from a psychiatric disorder, is characterized by elevated secretion of ACTH and CORT that occurs in response to CRH hyperactivity. In the remainder of this review, we will discuss studies of previously identified human polymorphisms in the CRH pathway that are associated with the occurrence of psychiatric disorders (Table 3). 

### 4.1. CRH

Smoller and colleagues performed one of the first genetic association studies of *CRH* by studying children from families in which the parents were diagnosed with panic disorders [115]. They specifically focused on behavioral inhibition in children as a strong predictor of later development of anxiety disorders. They identified an allele linked to the *CRH* locus (173 bp in the dinucleotide repeat marker CRH-PCR1) that negatively associated with behavioral inhibition, particularly in children whose parents had panic disorder. A subsequent study identified 3 single nucleotide polymorphisms (SNPs) in the *CRH* gene, one of which is in the coding region, and a haplotype comprising SNPs that also associated with behavioral inhibition (Table 3) [116]. Although the sample sizes in these studies are relatively small and there exist potential for Type I or Type II errors, they demonstrate an important role for the *CRH* gene in mediating anxiety-like behaviors in humans. It is suggested from these studies that children with these SNPs in the CRH gene are less likely to display behavioral inhibition and thus, are at a decreased risk for developing anxiety disorders later in life.

### 4.2. CRHR1

The most studied SNPs in the *CRHR1*gene are a T rare allele in rs7209436, an A rare allele in rs110402, and a T allele in rs242924, which also form the *CRHR1 *T-A-T haplotype. Adults with a history of childhood abuse that were homozygous for either of the rare alleles had the lowest Beck Depression Inventory (BDI) scores for depression, while those homozygous for the common allele had the highest BDI scores, with the heterozygotes having BDI scores in between the two [117]. Moreover, the *CRHR1 *T-A-T haplotypes were overrepresented in women with a history of childhood abuse that did not have MDD compared to women with no childhood abuse/no major MDD and women with childhood abuse and MDD. There was an additive effect of the rare alleles in this study [117,118]. Analysis of HPA axis activity in individuals with childhood abuse and the common allele SNPs of *CRHR1 *showed increased plasma CORT in response to the Dexamethasone/CRH test [119]. The potential protective role for the aforementioned SNPs has also been confirmed in males [120,121]. Further examination by Grabe and colleagues however, showed an increased risk rather than protective effects of this T-A-T haplotype [122] (Table 3). Taken together, the majority of data indicate that the rare allele SNPs and haplotypes in *CRHR1 *provide protection against the development of depressive symptoms when an individual has experienced early life stress. Thus, it is possible that the identified SNPs are involved in decreasing the activity of *CRHR1* to reduce CRH hyperactivity-driven maladaptive changes. An additional study presented evidence suggestive of a protective role for the T-A-T haplotype of the *CRHR1 gene.* The authors propose that the T-A-T haplotype may impair memory consolidation of the emotional effects of childhood abuse and thereby reduce risk of depressive symptoms later in life for women [121]. This is a likely inference as studies in rodents have demonstrated CRH involvement in memory formation and consolidation [32,123,124]. Further investigation of the rs110402 SNP has shown that T common allele carriers have a younger age-of-onset for their first depressive episode and experience more seasonal-related episodes of depression [125]. Functional magnetic resonance imaging (fMRI) data has demonstrated the MDD patients with the G allele in the rs110402 SNP have alterations in brain activity consistent with increased vulnerability to depression [16]. The use of fMRI in investigating the functional effects of CRHR1 polymorphisms provides for greater avenues of understanding how the CRH signaling pathways affect the generation of psychiatric disorders. 

In a depressed population of Mexican Americans, a G-A-G haplotype from SNPs rs1876828, rs242939, and rs242941 in the *CRHR1* gene increases treatment response to antidepressants, fluoxetine and desipramine [126]. Two studies have shown that in a group of Chinese patients, a G-G-T haplotype for the same SNPs increases the risk of developing major depression [127,128]. These studies demonstrate that genetic risk for depression depends on factors such as ethnicity and possibly geographic location. This substantiates the need to evaluate the interaction of genes and environment in studies of human diseases. 

Stress is associated with an increased risk of substance abuse. A sample of adolescents that had experienced a number of stressful life events and had common variants in *CRHR1* (CC rs187831 or A rs242938) demonstrated increased binge drinking [72] and increased risk of alcohol drinking behavior [129]. A subsequent independent analysis demonstrated that the more stressful life events experienced in adolescents homozygous for CC rs187831, the lower the age of onset for drinking [130]. Furthermore, adolescent stress with either genotype (CC rs187831 or A carrier rs242938) increased the risk for young adult alcohol drinking. In addition, CRH gene variations that differ in corticosteroid sensitivity were found to influence the risk for alcohol use and dependence in rhesus macaques [131]. Briefly, animals were genotyped for a single-nucleotide polymorphism disrupting a glucocorticoid response element, rh*CRH* - 2232 C > G. The effects of this allele on CSF levels of CRH, behavior and ethanol consumption were measured. Macaques carrying the G allele had lower CSF levels of CRH. Infant macaques carrying the G allele were more exploratory and bold, and in adolescent and adult male macaques, the G allele was associated with increased exploratory/bold behavior when responding to an unfamiliar male. Adults with the C/G genotype also exhibited increased alcohol consumption.

These studies indicate that there exists a gene x environment interaction for risk of alcohol abuse, as studies that examined the effect of the SNPs alone found no association [132,133]. Analysis of a different CRHR1 SNP (rs110402) in schizophrenia patients shows that the T allele interacts with a G allele in a CRHBP SNP (rs3811939) to double the risk of comorbid alcoholism in these patients. Individuals with these SNPs also show a higher CRH-R1/CRH-BP mRNA ratio, indicating an increase in CRH reactivity as the plausible cause [134]. Rodent studies have demonstrated that in alcohol preferring (msP) rats, there is an increase in CRH-R1 transcripts in the brain and an increased risk to reinstate alcohol-seeking after a foot-shock stressor [71]. These studies indicate that the human SNPs described may function to increase CRH activity mediated through CRH-R1, to increase stress-induced alcohol consumption. There is also evidence that low stress exposure increases suicidality in individuals with a rs4792887 (TT) genotype in the *CRHR1 *gene [135]. Studying the interaction of stress and polymorphisms in CRH pathway genes will enable better insight into the ability of stress to precipitate psychiatric diseases.

### 4.3. CRHR2

In depressed individuals, carriers of the G allele of the rs2270007 SNP in *CRHR2 *were slower to respond to antidepressant (citalopram) treatment at 4 weeks post-treatment [125] indicating a potential role for this SNP in altering HPA axis homeostasis and as a predictor of antidepressant treatment response.

A separate study provided evidence for an association between *CRHR2* SNPs and increased suicidal behavior in people with bipolar disorder [136].

### 4.4. CRHBP

The CRH binding protein sequesters CRH thereby preventing it from binding its receptors. Thus, reduced CRH-BP activity may cause CRH hyperactivity. The polymorphisms discussed below point to a decrease in expression or activity of CRHBP as the reason for the associated disorders. Patients with MDD that have a rs10473984 (TT) genotype in *CRHBP* show increase HPA axis activity, decreased response to antidepressant treatment, and an increased chance of relapse [35]. SNPs in *CRHBP* have also been associated with vulnerability for unipolar depression [137] and stress-induced alcohol craving (Table 3) [138]. Interactions between CRHBP and CRHR1 polymorphism have been observed to increase suicide attempts [139] and alcoholism [134] in schizophrenic patients (Table 3). Hyperactivation of CRH signaling pathways is certainly capable of exerting these effects based on the data reviewed in animal and human studies.

### 4.5. The Potential of CRH-Pathways Genetic Studies

CRH plays a critical role in adaptation to stress [140]. Finely regulated CRH expression and sensitivity is required for proper function and homeostasis of the HPA axis [141]. Chronic stress and early-life trauma can result in permanent disruptions in the CRH system and may lead to psychopathology in adulthood. The data summarized in Section 4 begins to lend support to the notion that genetic variation in CRH-related pathways contributes to psychiatric disorders in humans, and could serve as a potential target for therapeutic intervention. As each of these psychiatric diagnoses is likely to reflect divergent pathogenic mechanisms, the ability to personalize therapies based upon established risk-altering genotypes would be particularly valuable.

**Table 3:** Human gene variants in CRH pathways associated with psychiatric disorders.

GENE	Polymorphism	Associated Disorder	Effect	References
*CRH*	173 bp in the dinucleotide repeat marker CRH-PCR1	Behavioral inhibition	Risk promoting	[115,116]
	rs6999100 (CC)			
	rs6159 (GG)			
	rs1870393 (CC)			
*CRHR1 *	rs7209436 (TT) rs110402 ((AA) rs242924 (TT)	Depression with adverse early life experiences	Protective	[117,118,119,120,121]
	T-A-T haplotype			
*CRHR1*	T-A-T haplotype rs17689882	Depression with childhood physical neglect	Risk promoting	[122]
	rs16940674			
	rs16940665			
*CRHR1*	rs110402 (TT)	Depression onset and seasonal episodes	Risk promoting	[125]
*CRHR1*	rs110402 (GG)	Depression vulnerability	Risk promoting	[16]
*CRHR1*	G-G-T haplotype	Genetic susceptibility to major depression and response to antidepressant treatment	Risk promoting	[126,127,128]
	rs1876828 (GG)			
	rs242939 (GG)			
	rs242941 (TT)			
*CRHR1*	rs1876831 (CC)	Alcohol consumption with life stress	Risk promoting	[72,129,130]
	rs242938 (A)			
*CRHR1*	rs4792887 (TT)	Suicidality after low stress exposure	Risk promoting	[135]
*CRHR2*	rs2270007 (GG)	Decreased response to antidepressant treatment in depressed patients	Risk promoting	[125]
*CRHR2*	5-2-3 haplotype (allele 3 in GT)	Suicidal behavior in bipolar disorder	Risk promoting	[136]
*CRHBP*	rs10473984 (TT)	Remission and decreased depressive symptoms with citalopram treatment	Risk promoting	[35]
*CRHBP*	rs1875999 (TT)	Unipolar depression	Risk promoting	[137]
*CRHBP*	rs10055255 (TT)	Stress-induced alcohol craving and negative mood	Risk promoting	[138]
*CRHBP CRHR1 *	rs3811939 (GG)	Comorbid alcoholism in Schizophrenic patients	Risk promoting	[134]
	rs110402 (TT)			
*CRHBP CRHR1 *	rs1875999	Suicidal behavior in Schizophrenia	Risk promoting	[139]
	rs169400665			

## 5. Targeting the CRH Pathway for Therapy

The studies reviewed here demonstrate that abnormal CRH function may dysregulate HPA axis activity and produce maladaptive behavioral responses. Animal studies with CRH antagonists have shown both anxiolytic and antidepressant effects. Additionally, antidepressant treatment in depressed patients that normalizes HPA activity decreases the likelihood of relapse. In humans, CRH antagonists have been used in clinical trials to evaluate efficacy in treatment of MDD. A clinical trial in 20 patients with MDD demonstrated that the CRH-R1 antagonist, R121919, is able to decrease anxiety and despair in multiple inventories [142]. When the patients ceased taking R121919, their symptoms returned. Furthermore, this drug was safely tolerated by the patients, improved sleep [143] and did not impair normal brain activity, heart conductance, or HPA axis activity [144]. High doses of R121919 in control individuals elevated liver enzymes and resulted in discontinued production. Currently, only one other CRH receptor antagonists is undergoing clinical trials to evaluate efficacy in treating anxiety disorders ([clinicaltrials.gov;NCT01018992]). It should be noted that other placebo-controlled trials with CRHR1 antagonists have failed to show efficacy in the treatment of generalized anxiety disorder [145] or major depression [146]. The CRB-BP also served as a potential target for therapy, but much more research in needed to evaluate how it can be effectively targeted to reduce or elevate CRH activity depending on the disorder.

## 6. Conclusions

The summarized data presented in this review provide important insights into a role for CRH in mediating the maladaptive behaviors associated with major mood disorders and demonstrate the complexity of the underlying neurocircuitries involved in mediating CRH activity. In addition, these studies implicate components of the CRH system as potential therapeutic targets for interventions aimed at treating the negative behavior sequelae of mood disorders. 

The CRH-CRH receptor signaling pathway provides a consistent and robust series of translational studies linking basic neuroscience investigation in model systems to human psychiatric diseases. Initial behavioral and physiological experiments in rodents have reliably predicted that human variation in the CRH-CRH receptor and CORT pathways would influence the risk of neuropsychiatric disorders. In the future, these variants may serve as potential predictors of risk for the development of psychiatric illnesses and forecast individual responses to particular drug based treatments. 
